# Impact of the COVID-19 Pandemic in Spain in the Successive Pandemic Waves on Hemodialysis Patients and Healthcare Personnel

**DOI:** 10.3390/jcm12134337

**Published:** 2023-06-28

**Authors:** Sebastian Mas-Fontao, Blanca Miranda-Serrano, David Hernán, Raúl López, Paula Manso, Fabiola Dapena, Mº Luz Sánchez-Tocino, Jose Guerrero, Mónica Pereira, Damián Carneiro, Adriana Iglesias, Lola Piña, Elena Guerrero, Marta San Juan, Cristina Ledesma, Alicia González, Araceli Rossignoli, Concepción Pereira, Marina Burgos, Ana Mª Sacristán, Emilio González-Parra, María Dolores Arenas

**Affiliations:** 1 IIS-Fundación Jiménez Díaz, 28040 Madrid, Spain; smas@quironsalud.es (S.M.-F.); egonzalezpa@senefro.org (E.G.-P.); 2 Centro de Investigación Biomédica en Red de Diabetes y Enfermedades Metabólicas Asociadas (CIBERDEM), ISCIII, 28029 Madrid, Spain; 3 Fundación Renal Íñigo Álvarez de Toledo, 28003 Madrid, Spain; bmiranda@secardiologia.es (B.M.-S.); dhernan@friat.es (D.H.); rlopez@friat.es (R.L.); pmanso@friat.es (P.M.); fdapena@friat.es (F.D.); lsanchez@friat.es (M.L.S.-T.); jguerrero@friat.es (J.G.); mpereira@friat.es (M.P.); dcarneiro@friat.es (D.C.); aiglesias@friat.es (A.I.); lpina@friat.es (L.P.); eguerrero@friat.es (E.G.); msanjuan.miguelsanz@friat.es (M.S.J.); cledesma@friat.es (C.L.); agonzalezh@friat.es (A.G.); arossignoli@friat.es (A.R.); cpereira@friat.es (C.P.); mburgos@friat.es (M.B.); asacristan@friat.es (A.M.S.)

**Keywords:** chronic kidney disease, COVID-19, hemodialysis patients, healthcare personnel, mortality, symptoms

## Abstract

(1) Background: The impact of SARS-CoV-2 has been variable over the time course of the pandemic and in different populations. The aim was to analyze the impact of COVID-19 infection in a known population of hemodialysis (HD) patients and professionals in Spain at different times of the pandemic. (2) Methods: We conducted an observational, descriptive study with a follow-up from 3 March 2020 to 23 April 2022 (776 days), using in average of 414 professionals and 1381 patients from 18 HD units in Spain. The data from the positive PCR or the rapid antigen detection test (RADT) subject were analyzed and segmented into six periods (waves). (3) Results: Of 703 positive COVID-19 tests, 524 were HD patients (74.5%), and 179 were HD professionals (25.5%). Overall, 38% of staff and 43% of patients were affected. Differences were observed in regard to incidence (21% vs. 13%), mortality (3.5% vs. 0%), and symptomatology between the patients and professionals and throughout the pandemic. (4) Conclusions: COVID-19 severity varied during different pandemic waves, with a greater impact seen in the first wave. HD professionals and patients had similar infection rates, but patients had higher mortality rates. Community transmission was the primary route of infection.

## 1. Introduction

The SARS-CoV-2 coronavirus pandemic, which was first identified in the city of Wuhan (China) at the end of 2019, showed the first cases in Spain at the end of February 2020. From then until the spring of 2022, the pandemic showed a typical behavior in waves, specifically in the case of Spain; six were officially defined in the study period [1].

This disease known as coronavirus disease 2019, or COVID-19, has had a tremendous overall health impact, but it has particularly targeted the most clinically vulnerable populations. This includes patients on hemodialysis, given that, in general terms, this population was already more prone to respiratory tract infections [2], and mortality from sepsis was 50 times higher than in the general population [3]. Therefore, along with a higher propensity to infection, there are other particularities of this population, such as the presence of systemic inflammation [4], which leads to higher mortality due to viral infections in combination with a lower effectiveness of vaccines [5]. 

Several studies have been published in the last two years to study the impact it has had, including various on the Spanish population, covering different aspects of the symptomatology and treatment of hemodialysis patients [6,7,8]. In fact, our group previously described the impact of the pandemic on the implementation of vascular access in our centers [8]. 

Moreover, in addition to the observed consequences on infected renal patients, the impact of the pandemic on health professionals, although not as well described, has been no less significant [9], and throughout the different waves, it has become a challenge to maintain the activity in the units, given the shortage of staff due to the sick leave resulting from the infection among professionals [10,11]. 

According to published data from the COVID-19 registry of the Spanish Society of Nephrology, 8700 patients on renal replacement therapy have been infected by the SARS-CoV-2 coronavirus in the last 2 years in Spain, with a distribution in peaks or waves throughout the pandemic and a variable presentation by geographical areas attributed to the number of exposed inhabitants in each area [12]; however, the real number of patients exposed in this study is unknow.

In this study, we aimed to analyze the real incidence of COVID-19 infections in a known total population of patients and professionals in 18 hemodialysis units, its characteristics, and its impact throughout the different waves of the pandemic and in different geographical regions.

## 2. Materials and Methods

### 2.1. Design

We performed an observational, descriptive study with a follow-up from 3 March 2020 to 23 April 2022 (776 days) in the total number of hemodialysis (HD) patients seen and the total number of health personnel who attended them throughout the study (an average of 406 professionals and 1381 patients) from 18 dialysis units from foundation renal in 3 geographical regions of Spain (Madrid (9 centers, 980 patients), Galicia (5 centers, 211 patients), and Castilla-León (4 centers, 107 patients)). All dialysis patients (incident and prevalent) and professionals in these 18 centers were included.

Both the number of patients and workers has fluctuated over the different periods, ranging from 1326 to 1521 patients and from 365 to 442 professionals.

The data were segmented into 6 pandemic periods (waves), which were established according to the Spanish Ministry of Health: the first wave was from the first case reported (7 March 2020) to 31 May; the second wave was from 1 June to 31 December 2020; the third wave was from 1 December 2020 to 31 March 2021; the fourth wave, dominated by the circulation of the Alpha variant, lasted from 1 April to 30 June 2021; the fifth wave, in which the Delta variant was predominant, started on 1 June and ended on 20 November 2021; and the sixth wave took place from 21 November 2021 until the end of the analysis, on 23 April 2022, which is associated with the omicron variant. These dates were used for the present analysis [9]. A wave was defined as a very exponential increase in cases and a rapid decrease in the cases.

### 2.2. Patients and Professionals COVID-19 Diagnosis

All patients with suspected COVID-19 or who were close contacts were tested by either or both of two diagnostic tests for active infection: the rapid antigen detection test (RADT) and/or a viral RNA detection test by reverse transcriptase chain reaction (RT-PCR). The PCR test for SARS-CoV-2 consists of a nucleic acid extraction from nasopharyngeal swab samples and subsequent amplification by real-time RT-PCR, using ORF1ab and N genes as targets.

The study was approved by the IIS-Fundación Jiménez Díaz Ethics Committee (act n° 03/19) and was performed in accordance with the Declaration of Helsinki and the European Union Clinical Trial Directive. The patients and workers who were enrolled provided written informed consent.

### 2.3. Variables

Demographic and clinical data were obtained through a review of medical records. Variables included were age, sex, clinical symptoms, known existence of close contact, days from close contact to onset of symptoms, need for hospital admission, mortality, dialysis unit to which the patient belonged, region of the dialysis unit, patient vs. professional, professional category of the latter, date of PCR test positivity, and outcomes.

In the case of patients, the data were obtained from the electronic medical records, while in the case of employees, the data were collected through direct communication with the unit supervisors, together with the checks and follow-up by the human resources department, cross-checked with the social security registrations and deregistration’s.

### 2.4. Statistical Analysis

Graphs’ data are presented as medians and interquartile ranges (IQRs), except when otherwise specified. Graphs and corresponding statistical tests were conducted in R (v4.0.2; https://cran.r-project.org/, 22 June 2020) or GraphPad Prism v8 software (GraphPad Software Inc., La Joya, CA, USA). The normality of the data was assessed using the Kolmogorov–Smirnov test. Then the Mann–Whitney U test or the Kruskal–Wallis’s rank test was employed for binary and multiple comparisons, respectively. Contingency tables and χ^2^ were employed for categorical variables.

## 3. Results

### 3.1. Study Population: Patients and Professionals

A total of 1787 subjects (1381 HD patients and 414 healthcare professionals) were followed from the first cases of COVID-19 in Spain, 3 March 2020 through 23 April 2022, for 779 days.

The demographic characteristics of the HD patients are shown in Table 1. The mean age was 67.16 ± 15.3 year; a majority (64.8%) were male, and 27.5% were diabetics. Dialysis vintage was 66.2 (IC 95% 56.8–75.7) months. The most common etiology is diabetes mellitus, at 27.5%, follow by unknown causes, at 23.1%; glomerulonephropathy, at 15.2%; vascular origin, at 11%; tubulo-interstitial nephropathy, 8%; polycystic kidney disease, at 5.7%; and other causes, at 9.4%.

### 3.2. COVID-19 Prevalence on Patients and Professionals

Table 1 lists the main characteristics of the patients who had a positive diagnosis forCOVID-19, while Table 2 summarizes the characteristics of the professionals who were infected. Overall, the percentage of affected subjects was similar in both groups: 524 patients (38%) and 179 professionals (43.2%) had at least one positive diagnosis of COVID-19 by PCR or rapid antigen detection testing (RADT).

A total of 524 HD patients were infected during the six waves, although the incidence varied significantly over the course of the pandemic, from the first to the sixth wave: 130/1376 (9.4%), 96/1515 (6.3%), 43/1336 (3.2%), 10/1346 (0.74%), 34/1433 (2.4%), and 211/1521 (13.8%), respectively.

### 3.3. COVID-19 Symptomatology

**Figure 1:**
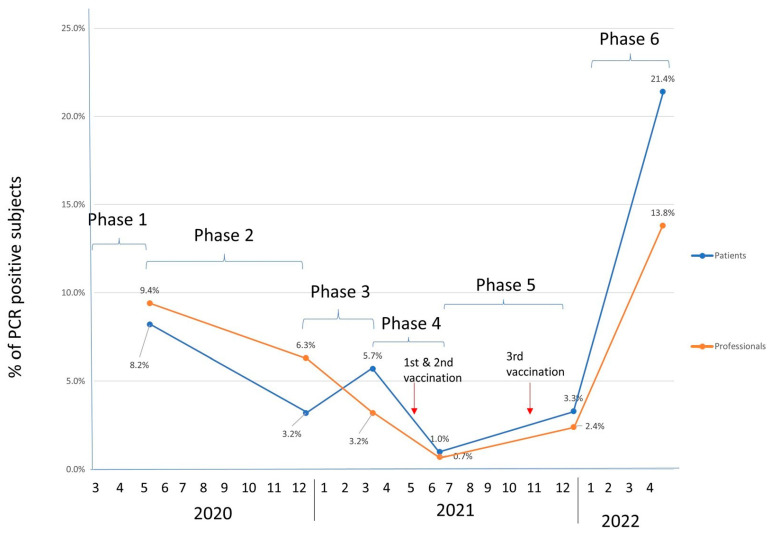
Percentage of COVID positives in patients (left) and professional (right) in each phase, expressed per total.

A total of 179 professionals were infected during the study period. The involvement of personnel varied over the six waves, being significantly higher in the sixth wave in relation to the overall number of personnel studied: 30/365 (8.2%), 14/440 (3.2%), 22/380 (5.7%), 4/386 (1.0%), 14/424 (3.3%), and 95/442 (21.4%) respectively. In total, 68.9% of nephrologists were infected (20/29), 52% of nurses (93/179) 42% of assistants (45/108), and 21.4% of other professionals (21/98). In overall terms, the percentage of infections in both groups was similar throughout the different phases (Figure 1).

### 3.4. COVID-19 Mortality

Total mortality in dialysis patients during the study period amounted to 54 subjects out of 1381, which represents 3.9% of the patients undergoing treatment in the various totals of dialysis centers and 10.3% of the total of the 524 patients infected. Among the centers belonging to the Foundation Renal network, mortality was concentrated in centers located in large cities (Madrid) and their outskirts, accounting for a total of 45 deaths out of a total of 54 (83.3%).

**Figure 2:**
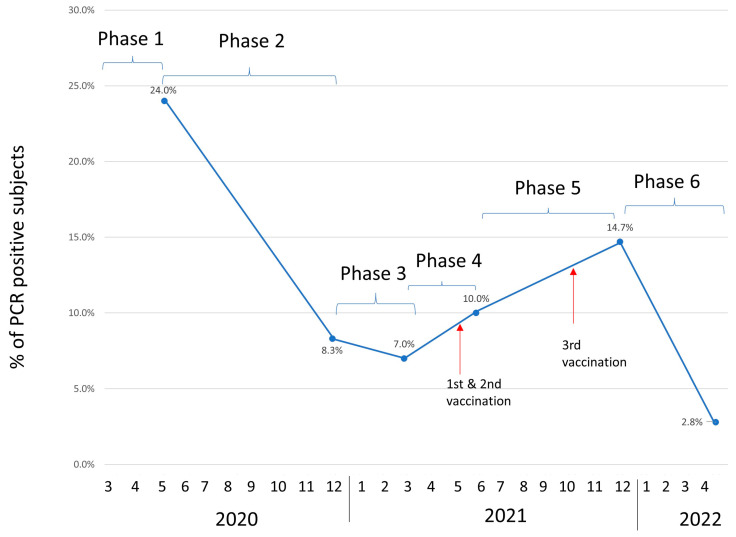
Percentage of deceased in relation to COVID-19-diagnosed patients. The blue line correspond to the “Percentage of exitus”.

Mortality, when viewed in relation to the number of patients on dialysis, varied greatly throughout the different phases, as shown in Figure 2. Thus, in Phase 1, it reached a rate of 0.22% of patients on HD, which accounted for 23.9% (31 out of 130) of the patients positively diagnosed in this first phase. Both the total number of infected patients and the number of deaths decreased in the successive phases: Phase 2 was 28.3% (8/96), and Phase 3 was 34.6% (3/43), corresponding to 0.05% to 0.02% of HD patients, respectively. This happened despite the strict preventive measures were implemented since March 2020 (Early Phase 1) [13].

In the fourth wave, the mortality rate reached a nadir. A rebound slightly occurred on Phases 5 and 6, but the mortality rate was still inferior to that in Phases 2 and 1.

In healthcare professionals, although the intensity of positives in the different waves was like that observed in patients, as mentioned, no deaths were reported among the different categories of staff that make up the Fundación Renal.

### 3.5. COVID-19 Transmission Route (Community vs. Center)

The detailed study of the route of infection of the different patients is summarized in Table 1. In most cases, the patient was not able to identify the route of transmission (72.9%). In the remaining cases, the most frequent route of transmission detected was the community route (22.9%, 120 patients) outside of the HD center as follows: 13 through friends, 87 through contact with relatives, 18 in the nursing home where they lived, and 2 during a period of hospitalization. Transmission in the center was suspected in 4.1%, 22 patients, with the majority of them suspected of being in the ambulance during transfer to the HD center or from the HD center (19); 2 infections happened in the dialysis treatment room; and 1 took place in the HD waiting room.

In the case of professionals, the majority of known infections was through family contact, followed by friends [7] or workplace in the hospital, not at the HD center [2]. In 131 cases, the origin could not be traced.

The known route of infection in patients varied from the first phase, where 55% of infections occurred during ambulance transport (11/21 patients), followed by infections in residences, families, and friends (42.8%; 9/21 patients) (Table 1), while in Phase 6, more than half of the infections with known route occurred through contact with family members (64/97; 65.9%). The other variable that was significantly associated with the route of infection, in addition to phase (Table 1), was age. Thus, older age (*p* = 0.02) was associated with infection from nursing-home and hospital patients, while younger age was associated with a higher percentage of infection from friends and relatives.

Among personnel, contact with family members was the most frequent known cause of infection across all waves (ranging from 100% (2/2) in the first wave to 77% (28/36) of cases), and no cases of contagion were detected at the centers.

## 4. Discussion

The present study has two main findings that may impact the care of hemodialysis patients with COVID-19. First, the 1-year mortality of hemodialysis patients with COVID-19 was high, and a majority of the deaths (70%) occurred after discharge. Thus, careful monitoring is required in the early post-COVID-19 period, and prophylactic measures (such as isolation of positive patients in separate rooms or in the same room, use of masks in the hemodialysis room or in the waiting room on a continuous basis, etc.) should be studied. The mid- and long-term impact of vaccines on antibody development should be monitored in order to develop guidance as to the use of booster doses in hemodialysis patients. The COVID-19 pandemic has lasted for more than two years, with a presentation in the form of infectious phases, colloquially called waves, characterized by an exponential growth and a similar decline of cases, spaced by periods of lower incidence. In this article, we describe the impact of these waves on 1381 HD patients (high risk individuals) [14] compared to the 414 healthcare professionals (low risk) who have cared for them over this time.

The most noteworthy result of this study is the uneven impact of the different waves on the population incidence, mortality, and symptomatology of COVID-19 infection, as well as the known route of infection between patients and professionals.

The numbers of HD patients and professionals affected have changed in parallel throughout the pandemic (Figure 1). Thus, in the first wave, nearly 10% of patients and 8.5% of professionals were affected, as is to be expected as a reflection of the great impact that COVID-19 had in the general population during this first wave in Spain [15], even after having taken early containment measures in dialysis units in anticipation of the impact the pandemic could have [16,17,18]. The number of positive patients decreased in subsequent phases, possibly due to better protective measures and, above all, to the early screening of all people in contact with them. This trend was reversed in the fifth phase and especially in the sixth phase, reaching an infection rate of 22% and 14% in patients and professionals, respectively, most likely due to the relaxation of outside-hospital protective measures and, probably, to the emergence of new variants due to mutations of the virus, such as omicron, in Spain in December 2021 [19]. SARS-CoV-2 omicron has been described as highly transmissible and to be spreading faster than any previous variant [19].

Mortality does not directly correlate with the number of infected patients, being much higher in the first phase, 23.9% of PCR positive+ patients; however, in the sixth phase, it was only 2.8% (Figure 2), even though the number of positives was three times higher (Figure 1). This mortality in HD patients is similar to that reported by other studies published during the first wave of the pandemic [7,20]. As previously mentioned, the appearance of the omicron variant has probably influenced this increase in cases without a subsequent increase in mortality, since this variant is more infectious and causes less severe symptoms than previous variants, although there has also been less immunity with vaccination in these strains [21,22]. On the other hand, the drastic reduction observed in mortality from the third phase onwards could also be explained by the introduction of preferential vaccination in this population, as well as by the personnel who take care of them (100% of them were vaccinated). Although the vaccines took only 9 months to manufacture, other work has already reported high efficacy, almost 100%, in preventing severe moderate disease and 70–95% efficacy in preventing infection in the general population [23]. Some studies show that immune response following vaccination in dialysis patients is almost comparable to controls with a high seroconversion rate (99%) but less antibodies concentrations than the general population in need of a booster [24,25,26]. It has already been shown with other vaccines, such as the Hepatitis B vaccine, that hemodialysis patients need higher and more frequent doses than the general population [27]. Despite this, in our study, we found a sharp decrease in mortality from the third wave onwards that could also be related to the vaccination of this population (1° doses of vaccination at the last of first phase and third doses during forth phase of pandemic).

Symptomatology has changed over time from the first phase, which was dominated by fever in the mildest cases to dyspnea in the most severe cases, similar to the first studies conducted during the first pandemic in hemodialysis patients [16,17,18] in which the symptoms evolved over time to more flu-like symptoms, possibly due to changes in the variants of the virus [28,29] and efficacy of vaccination.

The different impact of COVID-19 infection between renal patients and healthcare professionals in terms of both symptoms and mortality is striking; it shows the vulnerability of renal patients compared to the general population [30]. It also seems that the presence of other associated pathologies in renal patients may influence the increase in mortality [31]; despite this, a primary care study of 17,278,392 adults reported 10,926 COVID-19-related deaths. The most important factor associated with mortality was previous chronic kidney disease by itself [32]. The introduction of the vaccination pattern in Stages 4 and 5 seems to have a dramatic effect on mortality in the sixth wave (2.8%). In this study, 99.4% of patients were fully vaccinated. A recent study showed a decrease in mortality in HD patients after vaccination [33]. The impact of the different strains would also have an effect on infectivity and mortality; in particular, the omicron strain was associated with the increase in the number of infections observed in the sixth wave.

The change observed through different waves makes it necessary to consider updating the current protection and isolation recommendations for patients requiring renal replacement therapy in dialysis programs, adapting them to the current epidemiological context, which is radically different from that of the first wave. The possibility of being dialyzed in the same room as the negative patients but wearing an FPP2 mask during the entire transport, in the waiting room, and during the hemodialysis session is allowed. The staff caring for these patients will wear gloves, a gown, goggles, and an FPP2 mask [33]. On the other hand, knowledge of past infections gained through the surveying of healthcare professionals could be useful to avoid unnecessary quarantines, with better planning of healthcare resources [9].

Some limitations should be acknowledged. Although the population was closely followed, there are a large number of patients and professionals in whom the route of infection is unknown. One of the strengths of the study is the fact that it is a registry of a broad population group, which includes low-risk people (healthcare workers) and patients on hemodialysis (high risk), from the beginning of the pandemic, remaining in force and closely monitored since the first diagnoses until the sixth wave, so that the comparison between these patients and the people who care for them could give a good idea of the impact of the pandemic on our country in different populations.

## 5. Conclusions

The impact of COVID-19 infection in a hemodialysis population in Spain (patients and professionals) has varied throughout the pandemic, with greater severity in the first wave and a higher proportion of those affected, but with lesser severity, in the sixth wave. The proportion of infected professionals and patients has been similar but with higher mortality in patients. The most important route of infection has been community transmission in both populations.

## Figures and Tables

**Table 1:** Characteristics of HD patients and outcomes with confirmed COVID-19 by positive rapid antigen detection test (RADT) and/or a viral RNA detection test.

COVID-19 Positive	N	Phase 1	Phase 2	Phase 3	Phase 4	Phase 5	Phase 6	Test
Patients		(N = 130)	(N = 96)	(N = 43)	(N = 10)	(N = 34)	(N = 211)	Stat
Age	524	64.7 **77.0** 82.3	55.4 **65.0** 76.6	59.2 **69.0** 76.7	61.4 **68.5** 71.1	55.5 **70.0** 83.7	56.9 **70.0** 79.0	<0.01
Vaccination (1st and 2nd doses)	524	0% 0/130	0% 0/96	0% 0/43	90% 9/10	97.1% 33/34	97.6% 206	
Deceased (yes)	524	24% 31/130	8.5% 8/96	4.5% 3/43	10% 1/10	15% 5/34	3% 6/211	<0.01
Deceased unvaccinated vaccinated		24% 31/130 0% 0/130	8.5% 8/96 0% 0/96	4.5% 3/43 0% 0/43	100% 1/1 0% 0/9	100% 1/1 12.1% 4/33	20% 1/5 2.4% 5/206	<0.001
**Transmission route**	**524**							<0.001
**Center**	**4.1% 22**	**11**	**3**	**1**	**0**	**0**	**7**	
In center	2	0% 0/130	2% 2/96	0% 0/43	0% 0/10	0% 0/34	0% 0/134	
Waiting hall	1	0% 0/130	0% 0/96	0% 0/43	0% 0/10	0% 0/34	0.7% 1/134	
Ambulance	19	8.4% 11/130	1% 1/96	2.3% 1/43	0% 0/10	0% 0/34	4.4% 6/134	
**Outside center**	**22.9% 120**	**9**	**10**	**7**	**3**	**1**	**90**	
Residence	18	3.8% 5/130	0% 0/96	0% 0/43	0% 0/10	0% 0/34	9.7% 13/134	
Hospital	2	0% 0/130	0% 0/96	0% 0/43	0% 0/10	0% 0/34	1.5% 2/134	
Family	87	1.5% 2/130	10.4% 10/96	16.2% 7/43	30% 3/10	2.9% 1/34	47.7% 64/134	
Friends	13	1.5% 2/130	0% 0/96	0% 0/43	0% 0/10	0% 0/34	8.2% 11/134	
**Unknown**	**72.9% 382**	**110**	**83**	**35**	**7**	**33**	**37**	
Unknown	382	84.6% 110/130	86.4% 83/96	81.3% 35/43	70% 7/10	97% 33/34	27.6% 37/134	
**Symptoms**	**212**	77	45	17	5	7	61	<0.01
Cough	42 (19.8%)	26% 20/77	11.1% 5/45	17.65% 3/17	0% 0/5	14.25% 1/7	21.3% 13/61	
Dyspnea	37 (17.4%)	22% 17/77	13.3% 6/45	5.9% 1/17	60% 3/5	42.75% 3/7	11.5% 7/61	
Fever	59 (27.8%)	32.5% 25/77	46.6% 21/45	17.65% 3/17	0% 0/5	0% 0/7	16.4% 10/61	
Flu-like	47 (22.1%)	9% 7/77	13.3% 6/45	41.2% 7/17	40% 2/5	14.25% 1/7	39.3% 24/61	
GI	14 (6.6%)	9% 7/77	0% 2/45	5.9% 1/17	0% 0/5	14.25% 1/7	4.9% 3/61	
Others	13 (6.1%)	1.3% 1/77	11.1% 5/45	11.75% 2/17	0% 0/5	14.25% 1/7	6.6% 4/61	

COVID-19, SARS-CoV-2 PCR positive test; GI, gastrointestinal symptoms (diarrhea, etc.).

**Table 2:** Characteristics and outcomes of healthcare personnel with confirmed COVID-19 by positive rapid antigen detection test (RADT) and/or a viral RNA detection test.

COVID-19 Positive	N	Phase 1	Phase 2	Phase 3	Phase 4	Phase 5	Phase 6	Test
Professionals		(N = 30)	(N = 14)	(N = 22)	(N = 4)	(N = 14)	(N = 95)	Stat
Age	179	29.8 **34.5** 40.0	28.2 **37.5** 43.3	27.0 **33.0** 45.3	24.3 **29.0** 30.8	23.0 **42.0** 49.1	30.9 **38.0** 46.0	NS
Deceased (yes)	179	0% 0/30	0% 0/14	0% 0/22	0% 0/4	0% 0/14	0% 0/95	NA
**Transmission route**	**179**							<0.01
**Center**	0% 0	0	0	0	0	0	0	
**Outside center**	26.8% 48	2	4	3	1	2	36	
Hospital	2	0% 0/30	0% 0/14	0% 0/22	0% 0/4	0% 0/14	2.1% 2/95	
Family	39	6.6% 2/30	21.4% 3/14	13.6% 3/22	25% 1/4	14.2% 2/14	29.4% 28/95	
Friends	7	0% 0/30	0% 1/14	0% 0/22	0% 0/4	0% 0/14	6.3% 6/95	
**Unknown**	73.1% 131	28	10	19	3	12	59	
Unknown		93.3% 28/30	71.4% 10/14	86.3% 19/22	75% 3/4	85.7% 12/14	62.1% 59/95	
**Symptoms**	66	18	7	3	3	5	32	<0.01
Cough	6 (9%)	5% 1/19	0% 0/7	0% 0/3	0% 0/3	0% 0/5	6.2% 5/32	
Fever	25 (37.8%)	70% 13/19	57% 4/7	0% 0/3	0% 0/3	0% 0/5	25% 8/32	
Flu-like	23 (34.8%)	10% 2/19	29% 2/7	0% 0/3	0% 0/3	60% 3/5	50% 16/32	
GI	6 (9%)	10% 2/19	0% 0/7	66% 2/3	0% 0/3	20% 1/5	3.1% 1/32	
Others	6 (9%)	5% 1/19	14% 1/7	33% 1/3	0% 0/3	20% 1/5	6.2% 2/32	

COVID-19, SARS-CoV-2 PCR positive test; GI, gastrointestinal symptoms (diarrhea, etc.).

## Data Availability

All data from the study are available upon request.
